# Integrating Vision and Olfaction via Multi-Modal LLM for Robotic Odor Source Localization

**DOI:** 10.3390/s24247875

**Published:** 2024-12-10

**Authors:** Sunzid Hassan, Lingxiao Wang, Khan Raqib Mahmud

**Affiliations:** 1 Department of Computer Science, Louisiana Tech University, 201 Mayfield Ave, Ruston, LA 71272, USA; sha040@latech.edu (S.H.); krm070@email.latech.edu (K.R.M.); 2 Department of Electrical Engineering, Louisiana Tech University, 201 Mayfield Ave, Ruston, LA 71272, USA

**Keywords:** odor source localization, multi-modal robotics, large language models (LLMs), robot operating system (ROS)

## Abstract

Odor source localization (OSL) technology allows autonomous agents like mobile robots to localize a target odor source in an unknown environment. This is achieved by an OSL navigation algorithm that processes an agent’s sensor readings to calculate action commands to guide the robot to locate the odor source. Compared to traditional ‘olfaction-only’ OSL algorithms, our proposed OSL algorithm integrates vision and olfaction sensor modalities to localize odor sources even if olfaction sensing is disrupted by non-unidirectional airflow or vision sensing is impaired by environmental complexities. The algorithm leverages the zero-shot multi-modal reasoning capabilities of large language models (LLMs), negating the requirement of manual knowledge encoding or custom-trained supervised learning models. A key feature of the proposed algorithm is the ‘High-level Reasoning’ module, which encodes the olfaction and vision sensor data into a multi-modal prompt and instructs the LLM to employ a hierarchical reasoning process to select an appropriate high-level navigation behavior. Subsequently, the ‘Low-level Action’ module translates the selected high-level navigation behavior into low-level action commands that can be executed by the mobile robot. To validate our algorithm, we implemented it on a mobile robot in a real-world environment with non-unidirectional airflow environments and obstacles to mimic a complex, practical search environment. We compared the performance of our proposed algorithm to single-sensory-modality-based ‘olfaction-only’ and ‘vision-only’ navigation algorithms, and a supervised learning-based ‘vision and olfaction fusion’ (Fusion) navigation algorithm. The experimental results show that the proposed LLM-based algorithm outperformed the other algorithms in terms of success rates and average search times in both unidirectional and non-unidirectional airflow environments.

## 1. Introduction

Humans sense the external environment using sensory systems such as vision, olfaction, audition, etc. The data are then used for decision making within the environment. Similarly, a mobile robot can perceive the environment using artificial sensory devices like a camera, chemical sensor, microphone, etc. Of the sensory systems, olfaction was the first to evolve in organisms [1], allowing them to detect predators, food, potential mates, etc. [2]. However, the application of olfaction in robotics is not well studied. Robotic OSL is the technology that allows robots to localize an unknown odor source in the surrounding environment [3]. The technology is used in monitoring wildfires [4], chemical gas leaks [5], air pollution [6], underground gas leaks [7], identifying unexploded mines and bombs [8], locating hydrothermal vents [9], etc.

Advancements in robotics and autonomous systems have enabled the deployment of mobile robots to locate odor or chemical sources. Identifying the source of an unknown odor necessitates a proficient OSL navigation algorithm that directs the robot based on sensor readings [10]. Conventional OSL algorithms comprise animal-behavior-mimicking bio-inspired techniques, mathematical model-based engineering-based approaches, and machine learning-based strategies. Notable bio-inspired techniques include the moth-inspired algorithm, which emulates the mate-seeking behaviors of male moths [11], allowing a robotic agent to perform ‘surge/casting’ movements [12] to localize the odor source. Engineering-based approaches include the particle filter algorithm [13], infotaxis [14,15], stochastic mapping [16], etc. These methods update predictions regarding the odor source’s location based on olfactory observations. Lastly, machine learning-based OSL methods feature reinforcement learning [17] and deep supervised learning [18] techniques.

Most of the traditional OSL algorithms rely on olfactory (i.e., chemical and airflow) sensing to detect and navigate to the target odor source. However, methods that depend exclusively on olfactory sensing tend to underperform in environments where non-unidirectional airflow disrupts olfactory detection. The integration of vision and olfaction offers a great advantage to ‘olfaction-only’ systems. For example, fruit flies use a combination of vision and olfaction to locate colorful aromatic food [19]. They integrate olfactory information with visual landmark information to ‘see’ where the fruit aroma is coming from. This sensory integration allows them to precisely pinpoint the location of the fruit in their environment. Similarly, a robot equipped with both olfactory and vision-sensing abilities (such as a camera and a chemical sensor) and a navigation algorithm capable of effectively integrating these sensory modalities can more efficiently locate an unknown odor source in complex environments.

Humans often recognize visual objects in the surrounding environment and use relationship of those objects to the goal in making navigation decisions. A navigation system that tries to imitate such behavior needs to have several complex abilities—the ability to understand navigation objectives, the ability to detect objects from sensory inputs like vision, the ability to deduce contextual relations of those objects to the navigation goal, etc. Multimodal LLMs demonstrate state-of-the-art performance in reasoning over multiple sensory modalities like text, vision, and sound [20]. Compared to other expert systems, the motivation for using LLMs in this work is to utilize LLMs’ strong multi-modal semantic understanding and reasoning capabilities. However, applying these models in robotics introduces additional challenges, such as converting robot sensor readings into a format that can be processed by the LLMs, and subsequently translating the LLM’s textual outputs into actionable robot commands.

**Figure 1:**
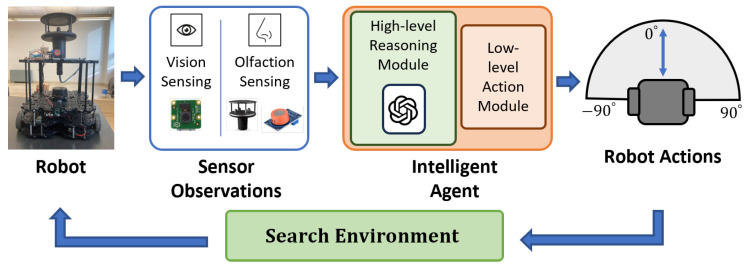
Flow diagram of the OSL system. The robot platform is equipped with a camera for vision and a chemical detector and an anemometer for olfactory sensing. The proposed algorithm utilizes a multi-modal LLM for navigation decision making.

The proposed OSL system is shown in Figure 1. The core of the system is an intelligent agent, which encodes vision and olfaction observations with a hierarchical navigation behavior selection instruction set for an LLM. The LLM then applies reasoning process over the multi-modal input and selects a high-level navigation behavior. Finally, a low-level action module translates the navigation behavior for the mobile robot. To validate the proposed algorithm, we conducted tests in a real-world environment where olfaction was challenged by non-unidirectional airflow, vision was challenged by obstacles, and multi-modal reasoning was challenged by environmental complexities.

The main contributions of this work can be summarized as follows:

1. Integrating vision and olfaction sensing to localize odor sources in complex real-world environments.
2. Developing an OSL navigation algorithm that utilizes the zero-shot multi-modal reasoning capability of a multi-modal LLM for OSL. This includes designing modules to process inputs to and outputs from the LLM model.
3. Implementing the proposed intelligent agent in real-world experiments and comparing its search performance to the rule-based Fusion navigation algorithm [21].

In the following sections of this paper, Section 2 includes a literature review of recent OSL algorithms; Section 3 reviews the details of the implemented OSL algorithm; Section 4 details the experiment setup and results; Section 5 includes limitations and future research directions; and finally, Section 6 presents the conclusions of the work. The code for this work can be found at https://github.com/SunzidHassan/24_LLM-OSL (accessed on 5 December 2024).

## 2. Related Works

### 2.1. Olfactory-Based Methods

Various organisms utilize olfaction to localize odor sources. This includes a bacterium navigating a gradient in amino acid or a lion tracking prey. Algorithms mimicking olfaction-based navigation behaviors of organisms is an effective approach in robotic OSL research.

Chemotaxis represents the simplest OSL strategy in biological organisms, where navigation relies solely on olfaction. For instance, bacteria demonstrate chemotaxis by altering movement based on odor concentration changes. They make fewer turns in the presence of an attractive chemical, resulting in straighter movement. Conversely, in the absence of a gradient or when moving away from higher concentrations, their default turning probability remains the same [22]. This straightforward approach allows single-celled organisms to navigate a gradient of appealing chemicals through a guided random walk. Nematodes [23] and crustaceans [24] also utilize chemotaxis-based OSL. Early OSL efforts focused on implementing such simple gradient-following chemotaxis algorithms. Typically, these methods used a pair of chemical sensors on plume-tracing robots, guiding them towards areas with higher concentration readings [25]. While early studies [26,27,28,29] validated chemotaxis in unidirectional flow environments, alternative OSL methods were proposed for complex non-unidirectional flow environments.

Anemotactic is a more sophisticated bio-inspired OSL method that uses both chemical and airflow senses for navigation. A wide variety of organisms, including moths [30,31,32], birds [33,34], etc., utilize this approach. Specifically, a prevalent moth-inspired method was developed by mimicking the mate-seeking behavior of male moths [35]. This was reinforced with additional bio-inspired search strategies in recent times, including zigzag, fuzzy inference, and multi-phase exploratory [36] search behaviors. Jin et al. [37] trained a neural network to predict gas flow patterns in an environment with obstacles, and combined it with the probabilistic source term estimation (STE) algorithm to localize gas sources in simulated and built environments. Ojeda et al. [38] utilized a predictive dispersion filament model to predict gas-hit maps from airflow data, and compared it to a measured gas-hit map to update the source probability distribution. Bio-inspired methods have also been applied in complex three-dimensional search areas [39,40]. Note that both chemical concentration and wind direction data are used in our proposed olfactory-based behavior.

Engineering-based methods differ from bio-mimicking algorithms by the utilization of mathematical models to estimate locations of odor sources. They involve discretizing the search area and learning the likelihood of each region containing the odor source. Algorithms used for generating such maps include particle filters, infotaxis [14,15], stochastic mapping [16], information-based search [41], source term estimation [42], partially observable Markov decision processes [43], reactive-probabilistic search [44], etc. After predicting the odor source location, robots are then guided to the source through path-planning algorithms like artificial potential fields and A-star [45,46].

Machine learning (ML)-based methods have achieved state-of-the-art performance in tasks of multiple data modalities, including vision, audio, and text. These methods have increasingly been applied for OSL tasks as well. In these types of methods, an artificial deep neural network processes robot sensor data and generates robot headings [10]. These models are trained using supervised or reinforcement learning methods. In supervised learning-based methods, an Artificial Neural Network (ANN) is trained as a classifier to predict the location of the odor source. For example, Kim et al. [18] trained a recurrent neural network (RNN) with a long short-term memory (LSTM) module to predict an odor source in simulation data. Bilgera et al. [47] used a convolutional LSTM model to detect an odor source in data measured from an anemometer and chemical sensors. Thrift et al. [48] trained a convolutional neural network (CNN) and a support vector machine (SVN) to classify directions of multiple odor sources. In reinforcement learning, an ANN is trained to generate action decisions to approach the odor source location. For example, Hu et al. [17] used a deterministic policy gradient actor–critic network for autonomous underwater vehicle (AUV) navigation. Wang et al. [49] developed an adaptive neuro-fuzzy inference system (ANFIS) for OSL in a simulated environment. Both of these methods were validated in simulated environments, highlighting the need for real-world implementations.

### 2.2. Vision and Olfaction Integration in OSL

The bio-inspired, engineering-based, and learning-based methods discussed above are ‘olfaction-only’. Olfaction-only approaches suffer if olfaction sensing is disturbed by non-unidirectional airflow, which is a common occurrence in real-world environments. Additionally, olfaction data are typically represented as the concentration level or detection rate of a chemical (e.g., ethanol). These representations inherently contain limited information about the location of the odor source. Thus, it is unclear if more complex algorithms can extract the ever-increasing amount of information from the olfaction data. Thus, it can be argued that the addition of vision sensing is the next paradigm in OSL research. Among the existing literature that has utilized vision sensing in OSL, Monroy et al. discussed using vision sensing with olfaction sensing for gas source localization [50]. They defined the odor footprint of some predefined objects using Web Ontology Language (WOL). They used the You Only Look Once v3 (YOLOv3) model for detecting those objects and looked up the odor footprint of those objects from the predefined knowledge base. The requirement of knowledge definition makes the model less scalable for complex environments.

In our previous work, we fused vision and olfaction for OSL using a custom-trained YOLOv6 model that directly detects visible plumes in the vision frame [21]. The algorithm was effective in localizing odor sources in real-world environments with obstacles and complex airflow. However, the vision model required visible odor plumes, and the algorithm followed olfaction-based navigation if odor plumes were invisible or obstructed. But even without visible odor plumes, vision data can still contain latent odor source location information that can help narrow search boundaries. For example, we may narrow our odor source search area to a restaurant without directly seeing the odor-emitting food. This information extraction requires the visual reasoning ability that multi-modal LLMs possess. This work aims to mitigate the limitations of previous vision and olfaction-based OSL models, i.e., to replace manual knowledge-based and supervised learning-based models with multi-modal reasoning-based models.

### 2.3. LLMs in Robotics

Large language models are a major milestone in the research of natural language processing (NLP). LLMs are specialized models for natural language generation [51]. These models are trained in a self-supervised learning approach, which negates the requirement for labeled training data. This allows the models to be trained on vast textual data on the internet. Additionally, it has been shown that there are similarities between the visual understanding of mammalian brains and the self-supervised learning approach [52] that is utilized by LLMs. The models are based on the transformer architecture, with a self-attention mechanism that allows them to learn complex interrelations in textual data [53]. LLMs exceed previous RNN-based language models due to emergent abilities, including chain-of-thought reasoning [54], instruction understanding [55], and in-context learning [56]. Notable examples of LLMs include BERT [57], GPT-3 [56], LLaMA [58], etc.

To further enhance theapplications of LLMs in embodied intelligence tasks, researchers are training these models with multi-modal data—text, image, audio, etc. These models are termed vision language models (VLMs) or multi-modal LLMs [59]. Unlike supervised vision classifiers, multi-modal LLMs are simultaneously trained with vision and language data. For example, the multi-modal LLM CLIP [60] is trained to minimize the distance of related images and text in a high-dimensional representation space. Training over massive multi-modal datasets allows these models to learn complex interrelationships among textual concepts and visual objects. This allows LLM-based robots to make zero-shot or few-shot reasoning over visual objects and states in a complex environment [61]. Thus, multi-modal LLMs are increasingly used in robotics tasks like generating robot action plans by reasoning over multi-modal sensor data [62].

In recent years, a richcollection of work has been published in the field of LLM-based robot navigation. These works can be broadly categorized into planning and semantic understanding models. Planning-based methods directly generate action decisions to guide the agent. Examples of such models include Clip-Nav [63], which utilizes an LLM for extracting key location phrases from the provided navigation objective, and uses CLIP VLM to ground the key phrases in the visual frame for navigation. $A^{2}$Nav [64] has five predefined actions, and separate navigators are trained for each of those actions. It utilizes the GPT-3 model for predicting actions, and the BERT model for aligning the predictions with the predefined actions. NavGPT [65] utilizes the GPT-4 model for zero-shot navigation in simulated indoor scenarios. VELMA [66] identifies landmarks from human-authored navigation instructions, and uses CLIP to ground them in a panoramic view of the robot. The model then generates a textual representation of the environment for textual command-based navigation. Semantic understanding models process sensor inputs, and the insights are then used to generate agent actions. Examples of such models include LM-Nav [67], which uses GPT-3 to translate verbal instructions into a series of textual landmarks. CLIP grounds the landmarks to a topological map, and a self-supervised robotic control model executes the physical actions. L3MVN [68] uses a language module to handle natural language instructions, generating a semantic map embedded with general physical world knowledge. Another module employs the semantic map to guide robotic exploration. ESC [69] conducts zero-shot object navigation by leveraging commonsense knowledge from pre-trained language models. It uses an LLM to ground objects and to deduce the semantic relationship of those objects in an indoor environment. Exploration techniques like ‘frontier-based exploration’ are used to navigate based on the semantic map. Conceptfusion [70] utilizes a multi-modal LLM to generate a multi-modal semantic map of the environment. The model can perform navigation using textual, visual, or audio cues.

### 2.4. Research Niche

The proposed LLM-based intelligent agent distinguishes itself from current LLM-driven robotic applications in two key ways. (i) First, our system differs in its input requirements. Rather than relying solely on visual observations, our model is designed to process both visual and olfactory sensory data. These multi-modal inputs provide the robot with a more comprehensive understanding of its environment, enabling richer interactions. (ii) Second, our model is purpose-built for a specific task: robotic OSL. Unlike generalized LLM-driven robots, which require vast amounts of training data and substantial computational resources, our system focuses on a specialized task. For example, training a general LLM-driven robot, such as Google’s RT-1 [71], for various object manipulation tasks involved data collection from 13 robots over 17 months: a costly process. In contrast, our system leverages pre-trained multi-modal LLMs.

## 3. Methodology

### 3.1. Problem Statement

The objective of robotic OSL is to develop a navigation algorithm that can subscribe to environment observations (i.e., state) from a mobile robot and process the state to generate action instructions for the robot to localize an unknown odor source in the robot’s surrounding environment. This process can be represented as

(1) $$a^{t}=F\left(s^{t}\right),$$

where $s^{t}$ is the robot observations at time *t*, and $a^{t}$ is the action output by the OSL function *F*.

**Figure 2:**
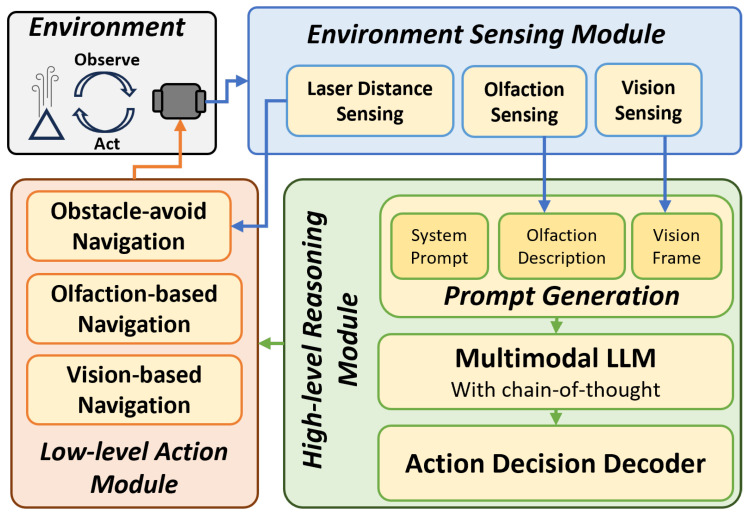
The framework of the proposed multi-modal LLM-based navigation algorithm. The three main modules are the ‘Environment Sensing’ module, ‘High-level Reasoning’ module, and ‘Low-level Action’ module.

Figure 2 illustrates the proposed robotic OSL framework. The algorithm has three primary modules: the ‘Environment Sensing’ module (Section 3.2), that processes robot sensory inputs; the ‘High-level Reasoning’ module (Section 3.3), that reasons over the input and decides a high-level navigation behavior; and the ‘Low-level Action’ module (Section 3.4), that translates those high-level behaviors into low-level actions that are executable by the robot.

### 3.2. Environment Sensing Module

**Figure 3:**
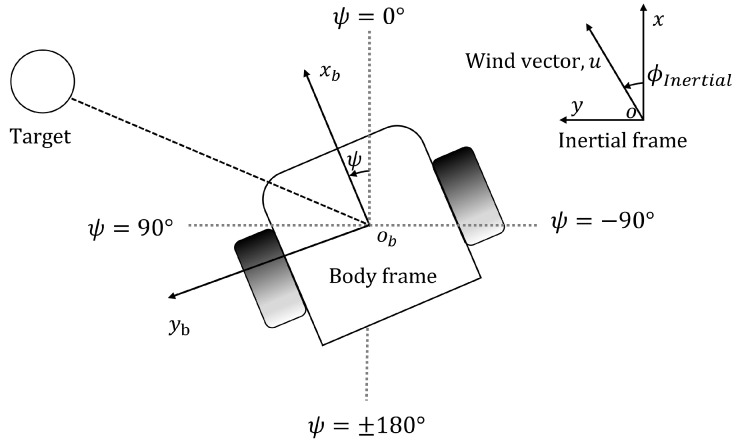
Robot notation. Robot position (x,y) and heading ψ are monitored by the built-in localization system. Wind speed *u* and wind direction are measured from the additional anemometer in the body frame. Wind direction in the inertial frame $\phi_{Inertial}$ is derived from robot heading ψ and wind direction in the body frame.

Figure 3 illustrates the environment sensing notations for this project. The agent is placed in an environment with an x−o−y inertial frame. The agent senses the environment in terms of its body frame $x_{b}-o_{b}-y_{b}$. Table 1 includes the parameter definitions and sensors. The mobile robot used in this work has a camera for visual detection, an anemometer and a chemical sensor for olfactory detection, and a laser distance sensor (LDS) for obstacle distance detection. The visual frame captured by the camera is the visual observation *p*. An anemometer senses wind speed, *u* m/s, and wind direction, $\phi_{b}$ degrees, in the body frame. The odor concentration ρ is expressed in ppm. At time *t*, the observed state by the robot is $s^{t}={\left[p,u,\phi_{b},\rho \right]}^{t}$. The sensors used in the real-world experimentation are discussed in Section 4.3.

### 3.3. High-Level Reasoning Module

The ‘High-level Reasoning’ module is the core of our proposed algorithm. The proposed algorithm uses a multi-modal LLM to perform zero-shot reasoning over multi-modal sensory inputs and decide high-level navigation behavior. Figure 2 shows the three main sub-modules: (1) prompt generation; (2) multi-modal reasoning; and (3) action decoding.

**Figure 4:**
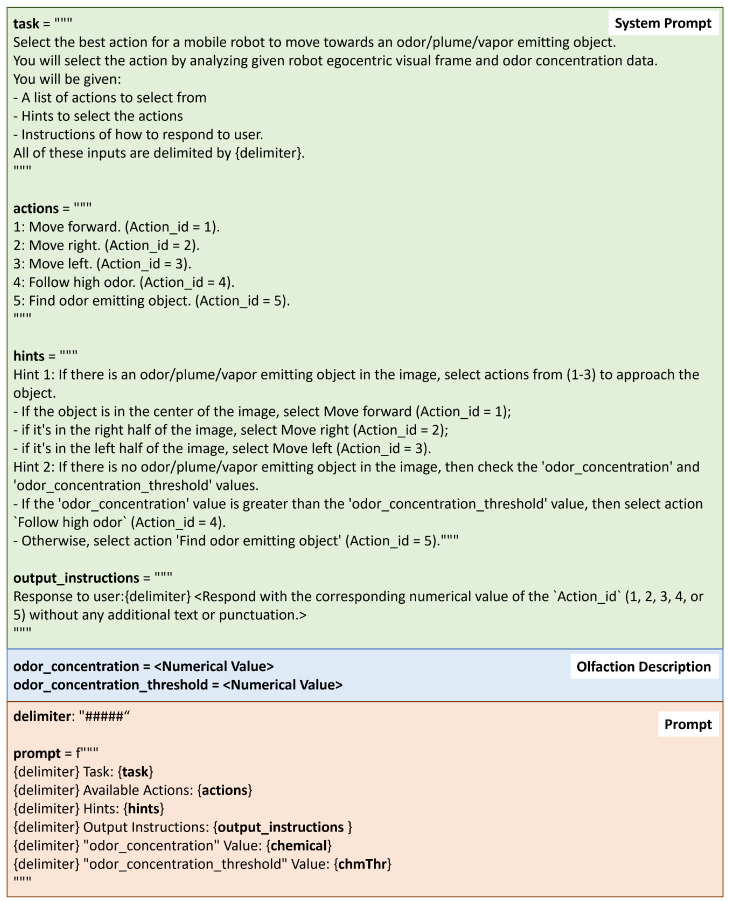
Implementation of the prompt. The system prompt includes the task, actions, hints and output instructions. The final prompt (orange box) includes the system prompt (green box) and the olfactory description (blue box).

Prompt generation is the first step in this module. Formulating effective prompts is crucial for LLM’s reasoning process. Figure 4 shows the prompt design, that includes the ‘system prompt’ and the ‘olfaction description’. Specifically, the system prompt includes the following:

- Task: Describes the objective and process the LLM should follow.
- Actions: Lists the actions available for the LLM to choose from.
- Hints: Guides the LLM to select appropriate vision-based or olfaction-based actions based on multi-modal reasoning.
- Output instruction: Directs the LLM to generate only the action without additional reasoning.

The olfaction description includes the current odor concentration ρ and odor concentration threshold as numeric values. The final prompt integrates all these instructions.

**Figure 5:**
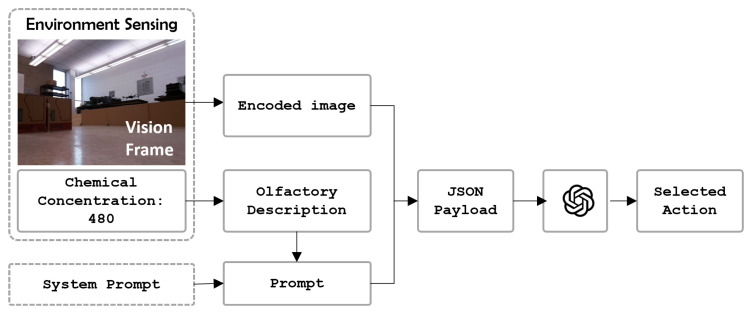
Querying the LLM with image and prompt. The input of the model is the visual frame and the prompt. The output of the model is the high-level action selection.

Figure 5 shows the process of querying the LLM. In this work, we employed GPT-4 as the multi-modal LLM. By default, requests are sent to GPT-4 as a JSON payload, where the image input *p* is encoded to text string using the default ‘base64’ function. Upon receiving the payload, GPT-4 then decodes this string back into an image format for processing. All these processes are encapsulated inside the GPT-4. That means that the GPT-4 is able to analyze the graphic information.

The LLM was instructed to use the chain-of-thought reasoning process [54] to capture logical coherence in multi-modal reasoning process over complex multi-modal sensory inputs. We should mention that chain-of-thought reasoning is a type of method in prompt engineering which studies how to ask questions (prompts) to LLMs. The goal of chain-of-thought reasoning is to help the LLM to decode a complex problem into several middle steps. Therefore, the most common usage of chain-of-thought reasoning is with LLMs. Based on the provided prompt, the multi-modal LLM model selects appropriate high-level ‘vision-based’ or high-level ‘olfaction-based’ navigation behaviors. The ‘system prompt’ contains instructions for the LLM to follow a hierarchical order while selecting the high-level navigation behaviors.

**Figure 6:**
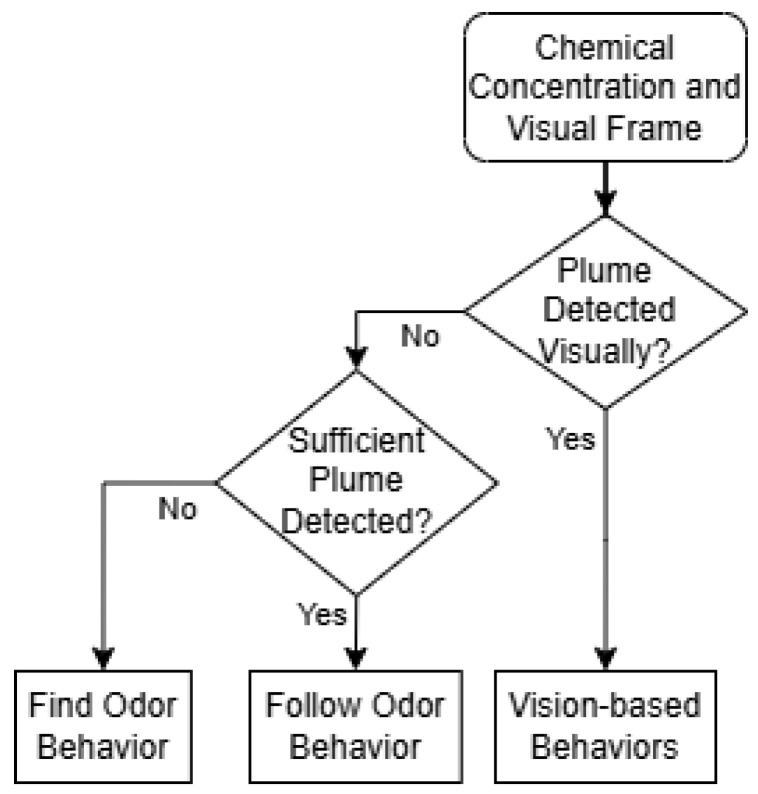
The flow diagram of the ‘High-level Reasoning’ module. It illustrates how the proposed LLM-based agent integrates visual and olfactory sensory observations to make high-level navigation behavior decisions.

Figure 6 illustrates the hierarchical reasoning strategy for one time step, which was modeled after human odor search behaviors, where vision and olfaction are used sequentially rather than simultaneously. Upon detecting an odor, vision is used first to locate the odor source. If visual reasoning fails to identify the source, the olfaction-based approach is employed to guide the robot toward the odor source. This decision-making process is repeated until the robot finds the odor source, moves out of the search area, or is out of time. Humans typically utilize vision to narrow down the odor source location. Based on ‘common sense’, humans can infer which objects within their visual field are likely to be odor sources. For instance, if we smell gas in a kitchen, we can deduce that the stove is a likely odor source. In this case, visual reasoning is utilized to pinpoint the odor source. Similarly, LLMs possess this kind of multi-modal ‘common sense’ reasoning, allowing them to deduce potential odor sources in their visual field.

The implemented reasoning module performs two primary visual reasoning tasks: (1) Finding odor source location information in the visual frame, i.e., odor source location or possible odor source direction; and (2) selecting appropriate ‘vision-based’ navigation behavior, i.e., forward, leftward, or rightward movement, to directly approach the odor source location. Otherwise, it analyzes the olfaction description and select either the ‘follow odor’ or ‘find odor’ navigation behavior. If a valid odor source object is later identified visually, the system switches back to vision-based navigation again. Lastly, the ‘action decoder’ extracts the output navigation behavior from the LLM and passes it to the ‘Low-level Action’ module.

### 3.4. Low-Level Action Module

The proposed algorithm has three high-level navigation behaviors: ‘obstacle-avoid’, ‘vision-based’, and ‘olfaction-based’ navigation behaviors. Of these, the ‘obstacle-avoid’ behavior is triggered directly if the LDS reading indicates that the robot is approaching an obstacle. The ‘vision-based’ and the ‘olfaction-based’ navigation behaviors are selected by the ‘High-level Reasoning’ module. The ‘Low-level Action’ module then translates those high-level behaviors into the low-level action vector

(2) $$a=[v_{c},\omega_{c}],$$

where $v_{c}$ is the linear velocity (m/s) and $\omega_{c}$ is the angular velocity (rad/s). The action vector is transmitted to and directly executed by the mobile robot.

‘Obstacle-avoid’: This behavior is activated when a nearby obstacle is detected by the onboard LDS. The ‘obstacle-avoid’ behavior directs the robot to navigate around the obstacle without deviating significantly from the direction the robot was following. Details of this navigation behavior are outlined in our previous paper [21].

‘Vision-based’: This is a class of behaviors that are selected and returned from the ‘High-level Reasoning’ module. The core strategy of vision-based navigation is to keep the detected target in the middle of the image. If the ‘High-level Reasoning’ module selects ‘vision-based navigation’ behavior, it returns one of three values for ‘behavior’—‘front’, ‘left’ or ‘right’—indicating if the robot should approach straight ahead or move towards the right or left to approach the odor source.

(3) $$\omega_{c}=\left\{\begin{array}{cc} 0 & \mathrm{if}\;\mathrm{action}\;=\;‘\mathrm{Front}’;\\ constant & \mathrm{if}\;\mathrm{action}\;=\;‘\mathrm{Left}’;\\ -constant & \mathrm{if}\;\mathrm{action}\;=\;‘\mathrm{Right}’. \end{array}\right.$$

Equation (3) is used by the ‘Low-level Action’ module for calculating linear and angular velocities, where the velocities are fixed as constant values. This means if ‘behavior’ is ‘front’, the robot will go straight ahead with a constant linear velocity without any angular velocity. If ‘behavior’ is returned as ‘right’ or ‘left’, the robot will execute both constant linear and angular velocity to rotate to the right or left to face the odor source.

**Figure 7:**
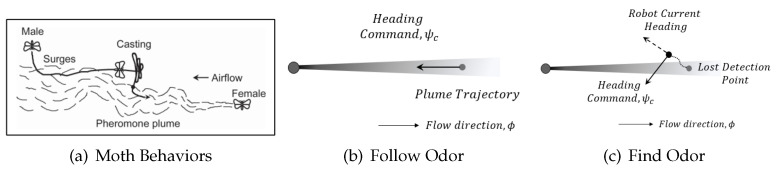
(**a**) Moth mate-seeking behaviors. This figure was retrieved from [73]. (**b**) Moth-inspired ‘surge’ and (**c**) ‘casting’ navigation behaviors.

‘Olfaction-based’: Finally, we utilize the moth-inspired ‘surge’ movement for implementing the high-level ‘follow odor’ behavior, and the ‘casting’ movement for implementing the high-level ‘find odor’ behavior [72]. Figure 7 shows the moth-inspired behaviors. In the ‘surge’ behavior, the robot moves upwind toward the odor source. In ‘casting’, the robot moves across wind to increase the likelihood of encountering odor plumes.

Equation (4) shows the target heading $\psi_{c}$ calculation for the two behaviors. Angular velocity $\omega_{c}$ is then adjusted to achieve the target heading $\psi_{c}$.

(4) $$\psi_{c}=\left\{\begin{array}{cc} \phi_{\mathrm{Inertial}}+180 & \mathrm{if}\;\mathrm{action}\;=\;‘\mathrm{Follow}\;\mathrm{Odor}’;\\ \phi_{\mathrm{Inertial}}+90 & \mathrm{if}\;\mathrm{action}\;=\;‘\mathrm{Find}\;\mathrm{Odor}’. \end{array}\right.$$

It should be noted that we used a reactive method due to its simplicity and lower computational cost compared to engineering-based methods (such as those involving mapping or memory). Adding mapping and memory would require more computational resources and could slow down the decision-making process. However, it should be noted that engineering-based methods can be implemented in the proposed ‘Low-level Action’ module without requiring any changes to the existing ‘High-level Reasoning’ module.

## 4. Experiment

### 4.1. Experiment Setup

**Figure 8:**
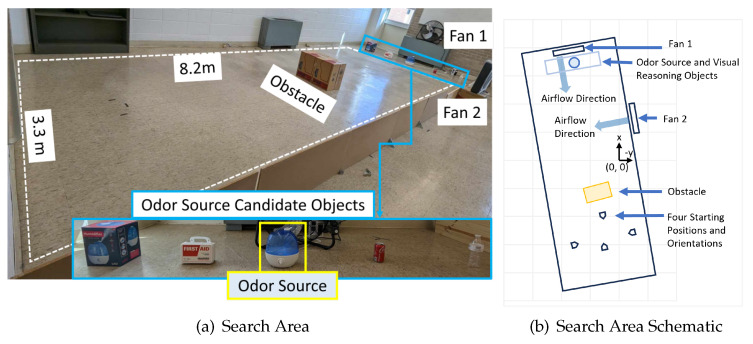
(**a**) Figure of the search area. The size of the search area is 8.2 m × 3.3 m. The odor source is a humidifier that generates ethanol plumes. An obstacle prevents vision of the plume initially and obstructs navigation. Two perpendicular electric fans are used to create unidirectional or non−unidirectional airflow. There are objects to test the visual reasoning capability of the LLM model. (**b**) Schematic diagram of the search area. We selected four different robot initial positions in the downwind area in the repeated tests.

The focus of the experiment is to test if the proposed navigation algorithm can reason over vision and olfaction sensory inputs to determine the actions to localize an unknown odor source. Figure 8 shows the search area used for the OSL navigation experiment. The search area has an obstacle in the middle to simulate complex indoor environments. There are multiple candidate odor source objects placed in the upwind area. The LLM must use reason to determine the correct odor source object from the candidate objects. The search area has an obstacle in middle. The purpose of the obstacle is to mimic constructed indoor environments, such as household environments, office environments, etc. The obstacle also initially prevents vision of the odor source. In order to succeed in localizing the odor source in this search area, the navigation algorithm must integrate vision and olfaction sensing and reason over them effectively. In this project, we define ‘unidirectional airflow’ as the condition when only one fan is used, and ‘non-unidirectional airflow’ as the condition when two perpendicularly placed fans are used. The odor concentration threshold is set to the background concentration, which is determined when the chemical sensor is not within the alcohol plume environment. The task is concluded successfully if the robot reaches within 0.8 m of the odor source within 120 s. It is important to note that this work primarily focuses on the design of the navigation algorithm. The key research question we aim to address is how to process multi-modal sensory inputs to compute robot actions that guide the robot toward the odor source. Source declaration in this work is defined by a distance threshold, meaning that reaching the threshold is considered as detecting the source.

### 4.2. Comparison of Algorithms

To validate the proposed OSL navigation algorithm, we have compared it with single-modality and multi-modality OSL navigation algorithms. Single-modality OSL navigation algorithms include the ‘olfaction-only’ and the ‘vision-only’ navigation algorithms. We also compared the performance of the multi-modal ‘Fusion’ navigation algorithm [21]. Unlike the visual reasoning-based navigation of the proposed LLM-based algorithm, the Fusion navigation algorithm utilizes a custom-trained ‘You Only Look Once version 7’ (YOLOv7) model to detect and then to navigate to the visible odor plumes. The primary goal of our experiments is to demonstrate that the proposed multi-modal OSL navigation algorithm outperforms single-modal algorithms, which include both olfaction-based and vision-based approaches. Most recent advancements in OSL are single-modal algorithms, primarily olfaction-based. For this study, we selected a representative olfaction-based algorithm, the moth-inspired method, due to the availability of its control code, which allowed us to implement it in our robotic agent.

The olfaction-only navigation algorithm comprises olfaction-based ‘surge’ and ‘casting’ behaviors with the ‘obstacle-avoid’ navigation behavior discussed in Section 3.4. The algorithm follows ‘obstacle-avoid’ behavior to navigate around the obstacles. In absence of obstacles, the algorithm tests the current odor concentration level against a threshold. If the detected plume concentration is below the threshold, the algorithm follows ‘casting’ behavior to maximize the chance of finding greater plume concentration. Otherwise, the algorithm follows ‘surge’ behavior to approach the upwind odor source.

In the vision-only navigation algorithm, the robot uses the ‘casting’, ‘vision-based’, and ‘obstacle-avoid’ behaviors discussed in Section 3.4. The algorithm follows ‘obstacle-avoid’ behavior to navigate around obstacles. Otherwise, the algorithm checks if there is any potential odor source cue in the visual frame. The algorithm follows ‘vision-based’ navigation if it finds visual cues towards the odor source. Otherwise, the algorithm moves perpendicular to the wind direction, resembling a ‘zigzag’ exploration movement, to increase the chance of detecting plume vision

The Fusion navigation algorithm utilizes a hierarchical control mechanism to select ‘surge’, ‘casting’, ‘obstacle-avoid’, or ‘vision-based’ navigation behaviors. In contrast to the ‘vision-based’ navigation behavior of the proposed LLM-based navigation algorithm, that uses zero-shot visual reasoning to identify a potential odor source object in the visual frame, the ‘vision-based’ navigation of the ‘Fusion’ algorithm is triggered if a custom trained YOLOv7 model detects a visible odor plume in the visual frame. In that case, the ‘vision-based’ navigation behavior tries to approach the visible plume directly.

The four navigation algorithms were tested in both unidirectional and non-unidirectional-airflow environments. For each environment, we used four distinct starting positions to demonstrate that our proposed method performs well from various initial positions and orientations, and four test runs were recorded from each starting position. Our experiments focus on analyzing how unidirectional and non-unidirectional flow environments impact the performance of each navigation algorithm, rather than the influence of the robot’s starting point. While only four trials were conducted per method for each starting point, each method was tested 16 times per environment (unidirectional or non-unidirectional), exceeding the commonly accepted trial range of 10–15. In our statistical analysis, we evaluate the effects of airflow environments and navigation methods, not the starting points. We conducted a total of 128 test runs, covering two airflow scenarios, four navigation algorithms, and 16 trials per scenario.

### 4.3. Robot Platform

**Figure 9:**
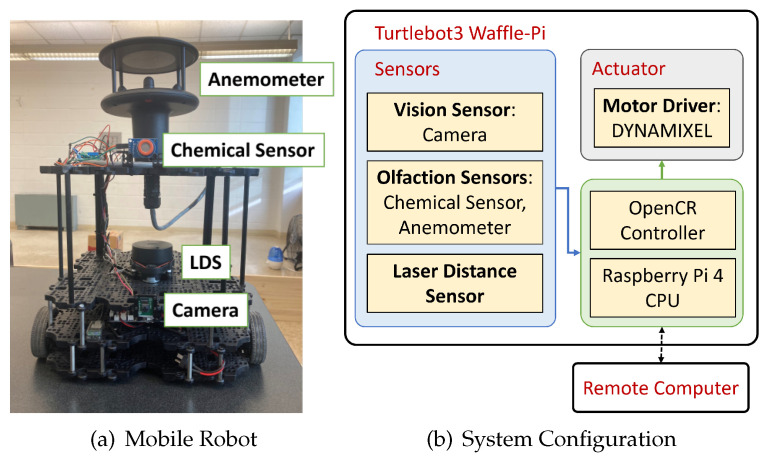
(**a**) The robot platform includes a camera for vision sensing and a chemical sensor and an anemometer for olfaction sensing. (**b**) The computation system consists of the robot platform and a remote PC. The dotted line represents a wireless link and the solid line represents a physical connection.

Figure 9a shows the robotic platform used in the real-world experiments. We used Turtlebot3 as the base for our robotic agent. In addition to the onboard vision sensors, we added an anemometer and a chemical sensor for olfactory detection. These sensors included the following:

- Camera: Raspberry Pi Camera v2, that can record 1080p video at 30 frames per second (FPS). This was used to capture the robot’s egocentric vision frame.
- LDS: LDS-02, that can detect 160–800 mm distance over 360 degrees. This was used to detect distances from obstacles.
- Anemometer: WindSonic, Gill Inc., that can sense 0–75 m/s wind over 360 degrees. This was used to record wind direction and speed.
- Chemical sensor: MQ3 alcohol detector, that can sense 25–500 ppm alcohol concentration. This was used to record odor concentration.

Figure 9b illustrates the system configuration, where the robot operating system (ROS) connects the robot platform to a remote computer over a local area network. The ROS publishes the sensor readings from the robot, which are subscribed to by the navigation algorithm running on the remote computer. The algorithm uses these readings to calculate and publish heading commands that the robot then executes. The robot subsequently collects a new set of sensor readings, and the cycle continues until it locates the odor source. The robot platform development is detailed further in our previous paper [74].

### 4.4. Sample Run

**Figure 10:**
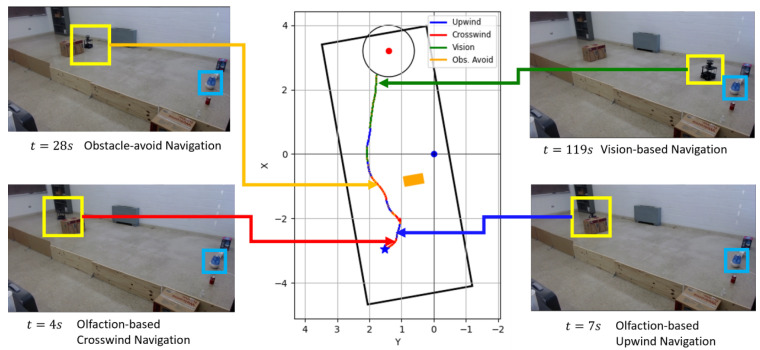
Trajectory graph of a successful sample run with the proposed multi-modal LLM-based OSL algorithm in unidirectional airflow environment. The navigation behaviors are color-separated. The obstacle is indicated by an orange box, and the odor source is represented by a red point with the surrounding circular source declaration region.

Figure 10 shows the robot trajectory and snapshots of a successful sample experiment run with the proposed algorithm in a unidirectional airflow environment. In this run, the robot was following ‘olfaction-based’ crosswind navigation at *t* = 5 s. At *t* = 7 s, it was sensing sufficient odor concentration and following ‘olfaction-based’ upwind navigation. At *t* = 28 s, the robot was following ‘obstacle-avoid’ navigation. Afterward, it followed ‘olfaction-based’ upwind and ‘vision-based’ navigation to reach the odor source at *t* = 119 s.

**Figure 11:**
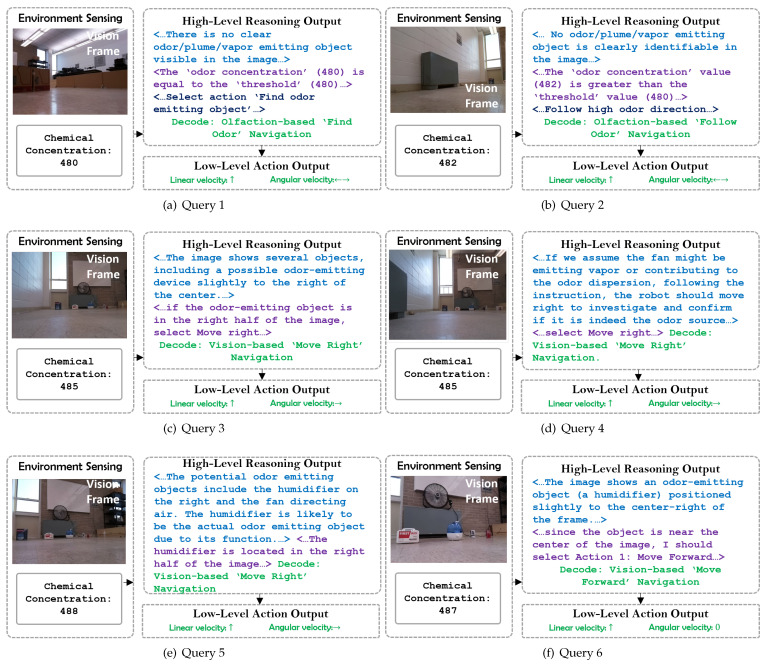
Examples of ‘environment sensing’ and ‘reasoning output’ by the GPT-4o model.

We then extracted the robot’s egocentric visual frames and chemical readings and used them to query the ‘High-level Reasoning’ module for navigation decisions with detailed reasoning output. Figure 11 illustrates prompt input and reasoning output by the ‘GPT-4o’ model from six time steps of the sample run. It should be noted that in this project, the LLM is instructed to navigate towards a single odor source in a zero-shot manner; no model training is involved in this process. The model does not provide a prioritized list of potential odor sources or any confidence scores. This is because in this work, the LLM is not commanded to choose which object is the odor source, but to choose which action the robot should select to approach the odor source.

In query 1, the model finds no possible odor source in the visual frame. Then, it checks the odor concentration and finds it to be less than the predefined threshold. Thus, the model outputs ‘find odor’ navigation behavior. In query 2, there was still no possible odor source in the visual frame. However, the model output was ‘follow odor’ navigation behavior as the odor concentration was above the threshold. In query 3, the model could not detect any clear odor source. But it detected the fan and deduced that approaching the fan could lead the robot closer to the odor source. In query 4, the model found a potential odor-emitting device in the right part of the visual frame and selected vision-based ‘move right’ navigation behavior. In query 5 the model found potential odor source objects, and decided to approach the humidifier based on its semantic understanding. In query 6, the model correctly detected the humidifier as the odor source and selected vision-based ‘move forward’ navigation behavior. Based on the navigation behaviors, the ‘Low-level Action’ module calculated proper linear and angular velocities for the robot. It should be noted that during experiment runs, we instructed the LLM not to generate textual output of the chain-of-thought reasoning to reduce the inference time. This brought the inference time down to under 3 s for most multi-modal queries.

### 4.5. Repeated Test Results

**Figure 12:**
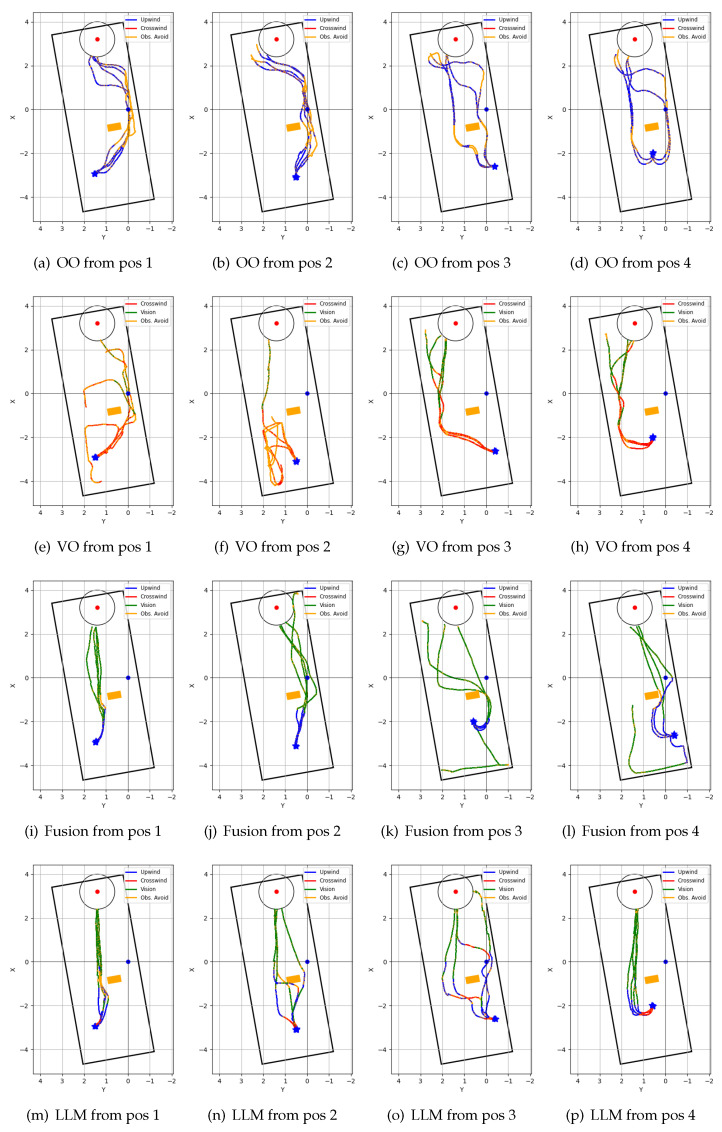
Robot trajectories of repeated tests in unidirectional airflow environment: (**a**–**d**) ‘olfaction-only’ (OO); (**e**–**h**) ‘vision-only’ (VO); (**i**–**l**) ‘vision and olfaction fusion’ (Fusion); and (**m**–**p**) ‘LLM-based’ (LLM) navigation algorithms.

**Figure 13:**
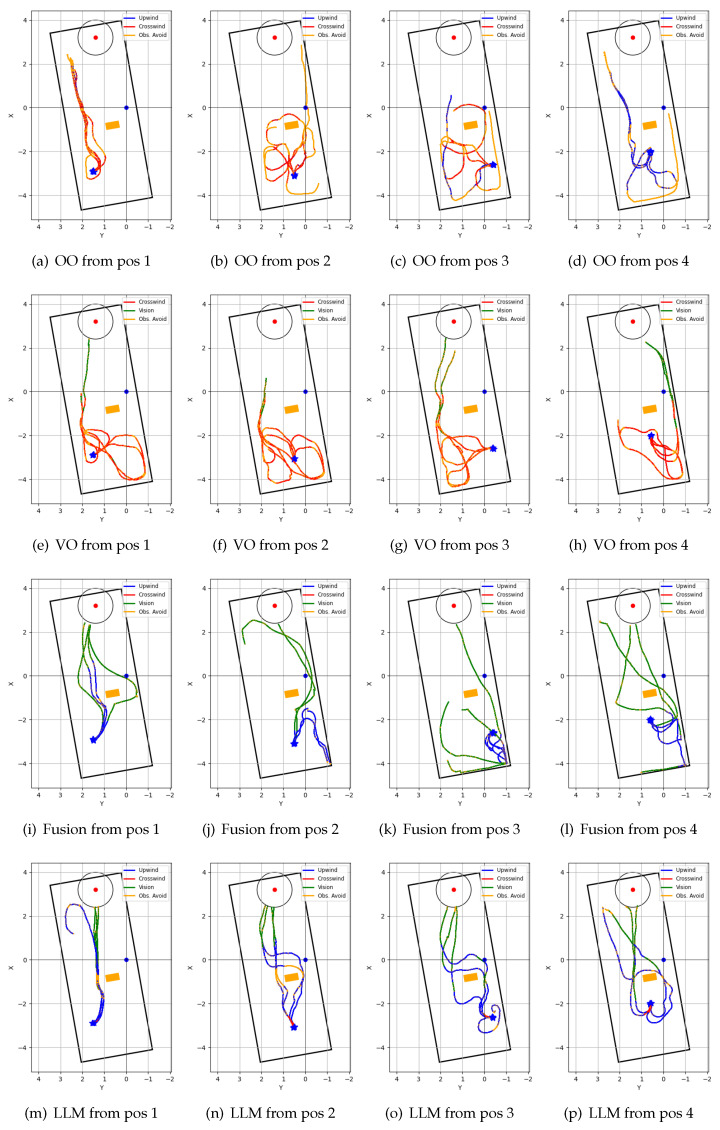
Robot trajectories of repeated tests in non-unidirectional airflow environment: (**a**–**d**) ‘olfaction-only’ (OO); (**e**–**h**) ‘vision-only’ (VO); (**i**–**l**) ‘vision and olfaction fusion’ (Fusion); and (**m**–**p**) ‘LLM-based’ (LLM) navigation algorithms.

Figure 12 shows the trajectories of the four algorithms in a unidirectional airflow environment, and Figure 13 shows the trajectories of the four algorithms in a non-unidirectional airflow environment. Each algorithm was tested from four fixed starting positions, and four trials were recorded from each starting position. Table 2 shows the performance comparison of the four navigation algorithms in a unidirectional airflow environment, and Table 3 shows the performance comparison in a non-unidirectional airflow environment.

In a unidirectional airflow environment, both the olfaction-only and the vision-only navigation algorithms performed poorly compared to the Fusion and proposed LLM-based navigation algorithms in terms of both mean search time and mean traveled distance. The proposed navigation algorithm performed better than all other algorithms in terms of success rate and mean search time. In a non-unidirectional airflow environment, the olfaction-only navigation algorithm failed to localize the odor source in all trial runs. The proposed navigation algorithm again outperformed other algorithms in terms of mean search time, mean distance traveled, and success rate.

While the olfaction-only navigation algorithm had a 62.5% success rate in unidirectional airflow, the success rate went down to 0% in the non-unidirectional airflow environment. The algorithm relies upon sufficient odor concentration detection and upon the assumption that the odor source is in the upwind direction. Complex airflow from multiple directions affects both of these aspects: it can dilute odor concentration, and non-unidirectional airflow from multiple directions can prevent OSL by upwind navigation.

The vision-based algorithm can only navigate towards the odor source if it is within its visual frame. The algorithm utilized crosswind movement, that resembles ‘zigzag’ like exploration movement perpendicular to the wind direction. This allowed the model to acquire initial plume vision in a unidirectional airflow environment. However, the algorithm often became sidetracked and lost plume vision while avoiding obstacles in the environment. This resulted in a 50% success rate. However, in a non-unidirectional airflow environment, the casting movement resulted in chaotic exploration of the environment. Thus, the success rate of the algorithm dropped down to 12.5%.

Both the Fusion navigation algorithm and the proposed LLM-based navigation algorithm utilize both vision and olfaction for localizing the odor source. Without proper visual cues, both of these algorithms follow olfaction-based crosswind movement to find the odor, and olfaction-based upwind movement to approach the odor source. Thus, their performance dropped in non-unidirectional airflow environments compared to unidirectional airflow environments. The Fusion navigation algorithm utilizes a deep learning-based vision model and follows a visible odor plume. In contrast, the proposed LLM-based navigation algorithm can reason over the vision frame to deduce the possible odor source direction. Thus, it can follow efficient vision-based navigation even without clearly discerning visible odor plumes or odor sources. In a unidirectional airflow environment, the proposed algorithm outperformed the Fusion algorithm in terms of both average success rate (100% vs. 75%) and average search time (80.3 s vs. 84.2 s). In a non-unidirectional airflow environment, the proposed multi-modal LLM-based navigation algorithm far exceeded the performance of the Fusion navigation algorithm in terms of average success rate (75% vs. 50%), average travel time (85.3 s vs. 97.7 s), and average traveled distance (6.4 m vs. 7.1 m).

**Figure 14:**
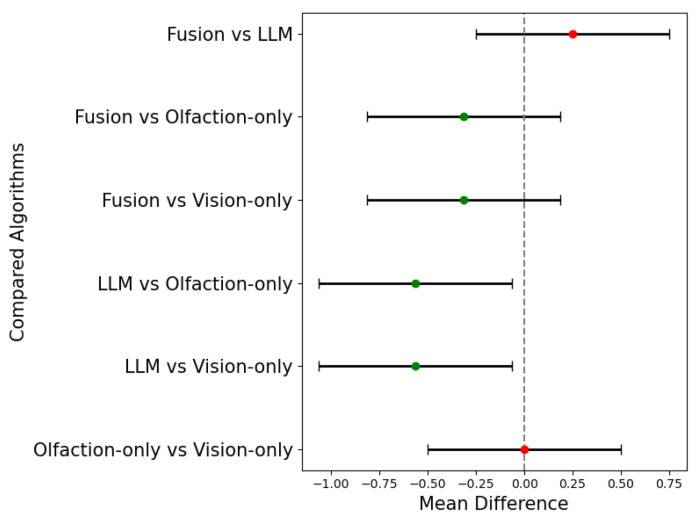
Mean differences of success rates of the four navigation algorithms. The positive differences are statistically significant at family-wise error rate (FWER) of 5%.

Figure 14 shows the Tukey’s honestly significant difference test (Tukey’s HSD) results among the success rates of the four algorithms. In the six one-to-one comparisons, the null hypothesis, $H_{0}$, states that the difference in the mean success rates of the two algorithms is not statistically significant with FWER of 5%. The results show that the null hypothesis is not rejected for comparisons with similar sensory modality algorithms, i.e., olfaction-only vs. vision-only and Fusion vs. LLM-based navigation algorithms. However, the differences are statistically significant for the comparison among mixed-modality algorithms. This indicates that the success rates of multi-sensory-modality-based navigation algorithms are statistically superior to the single-sensory-modality-based navigation algorithms.

## 5. Limitations and Future Work

One important limit for the LLM-based agent is the inference time. In our application, the inference time is three seconds. This can be reduced by using a smaller LLM. Secondly, our evaluation field is on a small scale. This field is sufficient to validate our proposed work, but a larger search area is required to test real-world-imitating applications. In the future, the 2-D OSL method discussed in this paper can further be extended to 3-D OSL scenarios using drones in larger search areas. Experiments can also be extended to actual environments, such as office or household environments. Reasoning-based vision processing can also be used to localize odor sources in 3-D spaces after initial plume recognition. The single-agent OSL discussed here can also be extended to multi-agent odor source localization or monitoring tasks over a larger area in the future. Example applications of multi-agent 3-D OSL include monitoring wildfire outbreaks in large forests or locating chemical leak sources in an indoor setting. Semantic search is another future direction, where next-generation olfaction sensors could detect the type of odor (e.g., chemical leak, food odor). In this case, the ‘High-level Reasoning’ module could search for specific odor sources in the visual field based on the detected odor type. Additionally, a more sophisticated anemotactic method could be implemented in the ‘Low-level Action’ module for more effective olfactory-based navigation. A more sophisticated source declaration algorithm that can rank potential odor sources could be incorporated with this model. Finally, reliance on LLM-based reasoning could be minimized by generating a semantic representation of the environment. In this approach, the LLM would only be queried when the robot senses unexplored parts of the environment. The semantic representation could then be used by alternative control mechanisms, such as reinforcement learning, for OSL navigation.

## 6. Conclusions

This paper presents a novel methodology to integrate vision and olfaction sensing in robotic OSL. The dual-modality integration allows the localization of odor sources even if olfaction or vision sensing is disrupted by environmental complexities. The innovation of this paper is the utilization of multi-modal LLM for zero-shot OSL navigation reasoning. We introduced a ‘High-level Reasoning’ module that generates a multi-modal prompt from robot sensor readings. This prompt is used to query the multi-modal LLM. The reasoning output of the LLM is then decoded and passed to the ‘Low-level Action’ module. The module then calculates commands that can be executed by the robot. To validate the performance of the proposed algorithm, we implemented the algorithm in a real-world environment. The environment’s non-unidirectional airflow challenges olfaction sensing, and obstacles and odor source candidate objects that challenge visual reasoning. We compared the performance of the proposed algorithm to single-sensory-modality-based ‘olfaction-only’ and ‘vision-only’ algorithms, and multi-sensory-modality-based ‘Fusion’ navigation algorithms.

- Our proposed method is a multi-modal navigation algorithm that integrates olfactory and visual sensors. Unlike single-modal algorithms, such as the moth-inspired navigation, our approach leverages visual inputs to enhance performance. For instance, when the robot visually identifies the odor source, it can approach it directly, significantly improving the success rate in locating odor sources in both unidirectional and non-unidirectional flow environments. Moth-inspired navigation relies primarily on wind measurements, achieving high success rates in unidirectional flows (10/16 in Table 2) but struggling in non-unidirectional flows (0/16 in Table 3). In contrast, with the help of visual detection, our method demonstrates robust performance across both scenarios. Additionally, in unidirectional search environments, the proposed method reduces the average search time by 18.1 s compared to the ‘olfaction-only’ navigation algorithm, and 14.9 s compared to the vision-based algorithm. In non-unidirectional search environments, the proposed method shortens the average search time by 5.37 s relative to the vision-based algorithm.
- Furthermore, compared to the rule-based vision and olfaction fusion algorithms (Fusion in Table 2 and Table 3), our approach incorporates the reasoning and semantic understanding capabilities of LLMs. This allows for more intelligent decision making beyond predefined rules. For example, when presented with an electrical fan (query 3 in Figure 11), the LLM can deduce that the odor source is likely near the fan—an inference unattainable by rule-based Fusion algorithms, which rely solely on recognizing visible odor plumes. As a result, compared to the Fusion navigation algorithm, the proposed method reduces the average search time by 3.87 s in unidirectional search environments, and by 12.49 s in non-unidirectional search environments.

The results also show that the success rates of the multi-sensory algorithms are significantly better than the success rates of single-sensory-modality-based algorithms. Overall, the results validate the proposed LLM-based vision and olfaction integration for OSL. In the future, this algorithm can be further expanded for multi-agent three-dimensional OSL environments for both indoor and outdoor OSL tasks. Furthermore, the method can be augmented with reinforcement learning methods to reduce computational costs.

## Figures and Tables

**Table 1:** Environment sensing parameters.

Symbol	Parameter
*p*	Visual observation
*u*	Wind speed
$\phi_{b}$	Wind direction in body frame
ρ	Odor concentration

**Table 2:** Comparison of search time (mean and std. dev.), traveled distance (mean and std. dev.), and success rates of the four tested algorithms in unidirectional airflow environment.

Navigation Algorithm	Search Time (s)		Traveled Distance (m)		Success Rate ↑
	**Mean** ↓	**Std. Dev.** ↓	**Mean** ↓	**Std. Dev.** ↓	
Olfaction-only	98.46	11.87	6.86	0.35	10/16
Vision-only	95.23	3.91	6.68	0.27	8/16
Fusion	84.2	12.42	6.12	0.52	12/16
Proposed LLM-based	**80.33**	4.99	6.14	0.34	**16/16**

**Table 3:** Comparison of search time (mean and std. dev.), traveled distance (mean and std. dev.), and success rates of the four tested algorithms in non-unidirectional airflow environment.

Navigation Algorithm	Search Time (s)		Traveled Distance (m)		Success Rate ↑
	**Mean** ↓	**Std. Dev.** ↓	**Mean** ↓	**Std. Dev.** ↓	
Olfaction-only	-	-	-	-	0/16
Vision-only	90.67	-	6.69	-	2/16
Fusion	97.79	4.69	7.08	0.53	8/16
Proposed LLM-based	**85.3**	5.03	**6.37**	0.31	**12/16**

## Data Availability

The raw data supporting the conclusions of this article can be found at URL: https://github.com/SunzidHassan/24_LLM-OSL (accessed on 5 December 2024).

## Abbreviations

The following abbreviations are used in this manuscript:

ANN	Artificial neural network
CNN	Convolutional neural netowrk
OSL	Odor source localization
LLM	Large language model
LSTM	Long short-term memory
Fusion	Vision and olfaction fusion
FWER	Family-wise error rate
Tukey’s HSD	Tukey’s Honestly Significant Difference
VLMs	Vision language models
WOL	Web Ontology Language

## References

[1] Purves D., Augustine G., Fitzpatrick D., Katz L., LaMantia A., McNamara J., Williams S. (2001). The Organization of the Olfactory System. Neuroscience.

[2] Sarafoleanu C., Mella C., Georgescu M., Perederco C. (2009). The importance of the olfactory sense in the human behavior and evolution. J. Med. Life.

[3] Kowadlo G., Russell R.A. (2008). Robot odor localization: A taxonomy and survey. Int. J. Robot. Res..

[4] Wang L., Pang S., Noyela M., Adkins K., Sun L., El-Sayed M. Vision and Olfactory-Based Wildfire Monitoring with Uncrewed Aircraft Systems. Proceedings of the 2023 20th International Conference on Ubiquitous Robots (UR).

[5] Burgués J., Hernández V., Lilienthal A.J., Marco S. (2019). Smelling nano aerial vehicle for gas source localization and mapping. Sensors.

[6] Fu Z., Chen Y., Ding Y., He D. (2019). Pollution source localization based on multi-UAV cooperative communication. IEEE Access.

[7] Chen Z., Wang J. (2017). Underground odor source localization based on a variation of lower organism search behavior. IEEE Sens. J..

[8] Russell R.A. (2004). Robotic location of underground chemical sources. Robotica.

[9] Wang L., Pang S., Xu G. 3-dimensional hydrothermal vent localization based on chemical plume tracing. Proceedings of the Global Oceans 2020: Singapore–US Gulf Coast.

[10] Jing T., Meng Q.H., Ishida H. (2021). Recent progress and trend of robot odor source localization. IEEJ Trans. Electr. Electron. Eng..

[11] Cardé R.T., Mafra-Neto A. (1997). Mechanisms of flight of male moths to pheromone. Insect Pheromone Research.

[12] López L.L., Vouloutsi V., Chimeno A.E., Marcos E., i Badia S.B., Mathews Z., Verschure P.F., Ziyatdinov A., i Lluna A.P. (2011). Moth-like chemo-source localization and classification on an indoor autonomous robot. On Biomimetics.

[13] Zhu H., Wang Y., Du C., Zhang Q., Wang W. (2020). A novel odor source localization system based on particle filtering and information entropy. Robot. Auton. Syst..

[14] Vergassola M., Villermaux E., Shraiman B.I. (2007). ‘Infotaxis’ as a strategy for searching without gradients. Nature.

[15] Luong D.N., Kurabayashi D. (2023). Odor Source Localization in Obstacle Regions Using Switching Planning Algorithms with a Switching Framework. Sensors.

[16] Jakuba M.V. (2007). Stochastic Mapping for Chemical Plume Source Localization with Application to Autonomous Hydrothermal Vent Discovery. Ph.D. Thesis.

[17] Hu H., Song S., Chen C.P. (2019). Plume Tracing via Model-Free Reinforcement Learning Method. IEEE Trans. Neural Netw. Learn. Syst..

[18] Kim H., Park M., Kim C.W., Shin D. (2019). Source localization for hazardous material release in an outdoor chemical plant via a combination of LSTM-RNN and CFD simulation. Comput. Chem. Eng..

[19] Frye M.A., Duistermars B.J. (2009). Visually mediated odor tracking during flight in Drosophila. JoVE (J. Vis. Exp.).

[20] Huang D., Yan C., Li Q., Peng X. (2024). From Large Language Models to Large Multimodal Models: A Literature Review. Appl. Sci..

[21] Hassan S., Wang L., Mahmud K.R. (2024). Robotic Odor Source Localization via Vision and Olfaction Fusion Navigation Algorithm. Sensors.

[22] Berg H.C. (2000). Motile behavior of bacteria. Phys. Today.

[23] Lockery S.R. (2011). The computational worm: Spatial orientation and its neuronal basis in *C. elegans*. Curr. Opin. Neurobiol..

[24] Radvansky B.A., Dombeck D.A. (2018). An olfactory virtual reality system for mice. Nat. Commun..

[25] Sandini G., Lucarini G., Varoli M. Gradient driven self-organizing systems. Proceedings of the 1993 IEEE/RSJ International Conference on Intelligent Robots and Systems (IROS’93).

[26] Grasso F.W., Consi T.R., Mountain D.C., Atema J. (2000). Biomimetic robot lobster performs chemo-orientation in turbulence using a pair of spatially separated sensors: Progress and challenges. Robot. Auton. Syst..

[27] Russell R.A., Bab-Hadiashar A., Shepherd R.L., Wallace G.G. (2003). A comparison of reactive robot chemotaxis algorithms. Robot. Auton. Syst..

[28] Lilienthal A., Duckett T. (2004). Experimental analysis of gas-sensitive Braitenberg vehicles. Adv. Robot..

[29] Ishida H., Nakayama G., Nakamoto T., Moriizumi T. (2005). Controlling a gas/odor plume-tracking robot based on transient responses of gas sensors. IEEE Sens. J..

[30] Murlis J., Elkinton J.S., Carde R.T. (1992). Odor plumes and how insects use them. Annu. Rev. Entomol..

[31] Vickers N.J. (2000). Mechanisms of animal navigation in odor plumes. Biol. Bull..

[32] Cardé R.T., Willis M.A. (2008). Navigational strategies used by insects to find distant, wind-borne sources of odor. J. Chem. Ecol..

[33] Nevitt G.A. (2000). Olfactory foraging by Antarctic procellariiform seabirds: Life at high Reynolds numbers. Biol. Bull..

[34] Wallraff H.G. (2004). Avian olfactory navigation: Its empirical foundation and conceptual state. Anim. Behav..

[35] Shigaki S., Sakurai T., Ando N., Kurabayashi D., Kanzaki R. (2017). Time-varying moth-inspired algorithm for chemical plume tracing in turbulent environment. IEEE Robot. Autom. Lett..

[36] Shigaki S., Shiota Y., Kurabayashi D., Kanzaki R. (2019). Modeling of the Adaptive Chemical Plume Tracing Algorithm of an Insect Using Fuzzy Inference. IEEE Trans. Fuzzy Syst..

[37] Jin W., Rahbar F., Ercolani C., Martinoli A. Towards efficient gas leak detection in built environments: Data-driven plume modeling for gas sensing robots. Proceedings of the 2023 IEEE International Conference on Robotics and Automation (ICRA).

[38] Ojeda P., Monroy J., Gonzalez-Jimenez J. (2024). Robotic gas source localization with probabilistic mapping and online dispersion simulation. IEEE Trans. Robot..

[39] Rahbar F., Marjovi A., Kibleur P., Martinoli A. A 3-D bio-inspired odor source localization and its validation in realistic environmental conditions. Proceedings of the 2017 IEEE/RSJ International Conference on Intelligent Robots and Systems (IROS).

[40] Shigaki S., Yoshimura Y., Kurabayashi D., Hosoda K. (2022). Palm-sized quadcopter for three-dimensional chemical plume tracking. IEEE Trans. Instrum. Meas..

[41] Hutchinson M., Liu C., Chen W.H. (2018). Information-based search for an atmospheric release using a mobile robot: Algorithm and experiments. IEEE Trans. Control. Syst. Technol..

[42] Rahbar F., Marjovi A., Martinoli A. An algorithm for odor source localization based on source term estimation. Proceedings of the 2019 International Conference on Robotics and Automation (ICRA).

[43] Jiu H., Chen Y., Deng W., Pang S. (2019). Underwater chemical plume tracing based on partially observable Markov decision process. Int. J. Adv. Robot. Syst..

[44] Luong D.N., Tran H.Q.D., Kurabayashi D. (2024). Reactive-probabilistic hybrid search method for odour source localization in an obstructed environment. SICE J. Control. Meas. Syst. Integr..

[45] Pang S., Zhu F. Reactive planning for olfactory-based mobile robots. Proceedings of the 2009 IEEE/RSJ International Conference on Intelligent Robots and Systems.

[46] Wang L., Pang S. Chemical Plume Tracing using an AUV based on POMDP Source Mapping and A-star Path Planning. Proceedings of the OCEANS 2019 MTS/IEEE SEATTLE.

[47] Bilgera C., Yamamoto A., Sawano M., Matsukura H., Ishida H. (2018). Application of convolutional long short-term memory neural networks to signals collected from a sensor network for autonomous gas source localization in outdoor environments. Sensors.

[48] Thrift W.J., Cabuslay A., Laird A.B., Ranjbar S., Hochbaum A.I., Ragan R. (2019). Surface-enhanced Raman scattering-based odor compass: Locating multiple chemical sources and pathogens. ACS Sens..

[49] Wang L., Pang S. An Implementation of the Adaptive Neuro-Fuzzy Inference System (ANFIS) for Odor Source Localization. Proceedings of the 2020 IEEE/RSJ International Conference on Intelligent Robots and Systems.

[50] Monroy J., Ruiz-Sarmiento J.R., Moreno F.A., Melendez-Fernandez F., Galindo C., Gonzalez-Jimenez J. (2018). A semantic-based gas source localization with a mobile robot combining vision and chemical sensing. Sensors.

[51] Chowdhary K. (2020). Natural language processing for word sense disambiguation and information extraction. arXiv.

[52] Nayebi A., Rajalingham R., Jazayeri M., Yang G.R. (2024). Neural foundations of mental simulation: Future prediction of latent representations on dynamic scenes. Adv. Neural Inf. Process. Syst..

[53] Vaswani A., Shazeer N., Parmar N., Uszkoreit J., Jones L., Gomez A.N., Kaiser Ł., Polosukhin I. (2017). Attention is all you need. Adv. Neural Inf. Process. Syst..

[54] Wei J., Wang X., Schuurmans D., Bosma M., Xia F., Chi E., Le Q.V., Zhou D. (2022). Chain-of-thought prompting elicits reasoning in large language models. Adv. Neural Inf. Process. Syst..

[55] Ouyang L., Wu J., Jiang X., Almeida D., Wainwright C., Mishkin P., Zhang C., Agarwal S., Slama K., Ray A. (2022). Training language models to follow instructions with human feedback. Adv. Neural Inf. Process. Syst..

[56] Brown T., Mann B., Ryder N., Subbiah M., Kaplan J.D., Dhariwal P., Neelakantan A., Shyam P., Sastry G., Askell A. (2020). Language models are few-shot learners. Adv. Neural Inf. Process. Syst..

[57] Devlin J., Chang M.W., Lee K., Toutanova K. (2018). Bert: Pre-training of deep bidirectional transformers for language understanding. arXiv.

[58] Touvron H., Lavril T., Izacard G., Martinet X., Lachaux M.A., Lacroix T., Rozière B., Goyal N., Hambro E., Azhar F. (2023). Llama: Open and efficient foundation language models. arXiv.

[59] Li C., Gan Z., Yang Z., Yang J., Li L., Wang L., Gao J. (2024). Multimodal foundation models: From specialists to general-purpose assistants. Found. Trends® Comput. Graph. Vis..

[60] Radford A., Kim J.W., Hallacy C., Ramesh A., Goh G., Agarwal S., Sastry G., Askell A., Mishkin P., Clark J. Learning transferable visual models from natural language supervision. Proceedings of the International Conference on Machine Learning.

[61] Shi Y., Shang M., Qi Z. (2023). Intelligent layout generation based on deep generative models: A comprehensive survey. Inf. Fusion.

[62] Wang J., Wu Z., Li Y., Jiang H., Shu P., Shi E., Hu H., Ma C., Liu Y., Wang X. (2024). Large language models for robotics: Opportunities, challenges, and perspectives. arXiv.

[63] Dorbala V.S., Sigurdsson G., Piramuthu R., Thomason J., Sukhatme G.S. (2022). Clip-nav: Using clip for zero-shot vision-and-language navigation. arXiv.

[64] Chen P., Sun X., Zhi H., Zeng R., Li T.H., Liu G., Tan M., Gan C. (2023). *A*^2^ Nav: Action-Aware Zero-Shot Robot Navigation by Exploiting Vision-and-Language Ability of Foundation Models. arXiv.

[65] Zhou G., Hong Y., Wu Q. Navgpt: Explicit reasoning in vision-and-language navigation with large language models. Proceedings of the AAAI Conference on Artificial Intelligence.

[66] Schumann R., Zhu W., Feng W., Fu T.J., Riezler S., Wang W.Y. Velma: Verbalization embodiment of llm agents for vision and language navigation in street view. Proceedings of the AAAI Conference on Artificial Intelligence.

[67] Shah D., Osinski B., Ichter B., Levine S. (2022). Robotic Navigation with Large Pre-Trained Models of Language. arXiv.

[68] Yu B., Kasaei H., Cao M. L3mvn: Leveraging large language models for visual target navigation. Proceedings of the 2023 IEEE/RSJ International Conference on Intelligent Robots and Systems (IROS).

[69] Zhou K., Zheng K., Pryor C., Shen Y., Jin H., Getoor L., Wang X.E. Esc: Exploration with soft commonsense constraints for zero-shot object navigation. Proceedings of the International Conference on Machine Learning.

[70] Jatavallabhula K.M., Kuwajerwala A., Gu Q., Omama M., Chen T., Maalouf A., Li S., Iyer G., Saryazdi S., Keetha N. (2023). Conceptfusion: Open-set multimodal 3d mapping. arXiv.

[71] Brohan A., Brown N., Carbajal J., Chebotar Y., Dabis J., Finn C., Gopalakrishnan K., Hausman K., Herzog A., Hsu J. (2022). Rt-1: Robotics transformer for real-world control at scale. arXiv.

[72] Farrell J.A., Pang S., Li W. (2005). Chemical plume tracing via an autonomous underwater vehicle. IEEE J. Ocean. Eng..

[73] Ishida H., Moriizumi T. (2002). Machine olfaction for mobile robots. Handbook of Machine Olfaction: Electronic Nose Technology.

[74] Hassan S., Wang L., Mahmud K.R. Multi-Modal Robotic Platform Development for Odor Source Localization. Proceedings of the 2023 Seventh IEEE International Conference on Robotic Computing (IRC).

